# Comparison of the differential expression miRNAs in Wistar rats before and 10 days after *S.japonicum* infection

**DOI:** 10.1186/1756-3305-6-120

**Published:** 2013-04-24

**Authors:** Hongxiao Han, Jinbiao Peng, Yang Hong, Min Zhang, Yanhui Han, Zhiqiang Fu, Yaojun Shi, Jinjun Xu, Jianping Tao, Jiaojiao Lin

**Affiliations:** 1 Shanghai Veterinary Research Institute, Chinese Academy of Agricultural Sciences, Key Laboratory of Animal Parasitology, Ministry of Agriculture, 518 Ziyue Road, Minhang, Shanghai, 200241, People’s Republic of China; 2 College of Veterinary Medicine, Yangzhou University, 12 East Wenhui Road, Yangzhou, Jiangsu, 225009, China; 3 Shanghai Public Health Clinical Center, Fudan University, Shanghai, 201508, China

**Keywords:** miRNAs, *Schistosoma japonicum* infection, Wistar rats

## Abstract

**Background:**

When compared to the murine permissive host of *Schistosoma japonicum*, Wistar rats are less susceptible to *Schistosoma japonicum* infection, and are considered to provide a less suitable microenvironment for parasite growth and development. MicroRNAs (miRNAs), are a class of endogenous, non-coding small RNAs, that impose an additional, highly significant, level of gene regulation within eukaryotes.

**Methods:**

To investigate the regulatory mechanisms provided by miRNA in the schistosome-infected rat model, we utilized a miRNA microarray to compare the progression of miRNA expression within different host tissues both before and 10 days after cercarial infection, in order to identify potential miRNAs with roles in responding to a schistosome infection.

**Results:**

Among the analysed miRNAs, 16 within the liver, 61 within the spleen and 10 within the lung, were differentially expressed in infected Wistar rats. Further analysis of the differentially expressed miRNAs revealed that many important signal pathways are triggered after infection with *S. japonicum* in Wistar rats. These include the signal transduction mechanisms associated with the Wnt and MAPK signaling pathways, cellular differentiation, with a particular emphasis on adipocyte and erythroid differentiation.

**Conclusions:**

The results presented here include the identification of specific differentially expressed miRNAs within the liver, lungs and spleen of Wistar rats. These results highlighted the function of host miRNA regulation during an active schistosome infection. Our study provides a better understanding of the regulatory role of miRNA in schistosome infection, and host–parasite interactions in a non-permissive host environment.

## Background

Schistosomiasis is one of the most prevalent and serious parasitic diseases worldwide, with nearly 200 million people at risk, the disease occurs in tropical and subtropical regions. The potential resistance to the drug praziquantel, together with the frequent re-infection of people in endemic areas, has stimulated the search for new control strategies for this disease. In order to plan strategies to provide alternative future therapies a better understanding of schistosome development and host–parasite interactions is required [1,2]. Two definitive hosts of schistosomes are mice (*Mus musculus*) and rats (*Rattus norvegicus*). Mice are permissive hosts of *Schistosoma japonicum* and support the full growth, development and sexual maturation of the parasite. In contrast, rats are less susceptible or semi-permissive, and do not provide a suitable microenvironment for parasite growth and development [3]. Many factors have been found to affect the life cycle of *S. japonicum* in rat hosts, such as the low survival rate of cercariae that penetrate through the skin, fewer schistosomula migrating successfully from the hepatic portal circulation into the mesenteric veins, and a lower rate of egg-laying and increased numbers of immature eggs in adult parasites [4]. Previous reports have indicated that the innate resistance of Wistar rats to *S. japonicum* may be related to the presence of natural antibodies against the parasite and other humoral and/or cellular immune responses [5,6].

MicroRNAs (miRNAs) are a class of endogenous, small noncoding RNAs that regulate gene expression, at transcription and post-transcriptionally, by the indirect regulation of transcription factors, and in the latter case through the induction of mRNA degradation or the direct inhibition of translation [7]. Therefore, miRNAs are very important in the control of developmental, physiological, and pathological processes, such as cellular differentiation, cell proliferation, and tumor generation [8-10]. The complex interaction between parasites and their hosts, such as drug resistance in parasites, may also be influenced by miRNAs [11,12]. A class of miRNAs has been found to regulate the promoter binding of the nuclear factor (NF)-kB p65 subunit in human cholangiocytes in response to *Cryptosporidium parvum* infection, and this may represent the regulation of epithelial antimicrobial defense [13]. However, few studies have investigated the differences in miRNA expression and its specific biological functions in hosts infected by parasites [14,15].

In the present study, a microarray technique was applied to analyze differences between infected and uninfected Wistar rats in terms of host miRNA expression within various tissues, with an aim to identify biological functions of differentially expressed miRNAs. The results provide novel comparative information to potentially define the functional significance of host miRNAs during a *S. japonicum* infection in the rat model. These findings will help to identify the molecular mechanisms associated with schistosome growth retardation within the semi-permissive rat host.

## Methods

### Animal challenge and tissue preparation

Wistar rats (8 weeks, male, ~150 g) were obtained from Shanghai Laboratory Animal Center, Chinese Academy of Sciences. All the animals were housed singly for 1 week before infection. The life cycle of *S. japonicum* (Chinese mainland strain, Anhui isolate) was maintained routinely in BALB/c mice and *Oncomelania hupensis* (snails) in the Shanghai Veterinary Research Institute. Food and water was available ad libitum. Sixty Wistar rats were randomly divided into six groups of ten for each infection and control group. The infection experiment was repeated in three independent biological replicates. Wistar rats were infected percutaneously with 2000 *S*. *japonicum* cercariae, respectively. The animals were sacrificed 10 days post-infection (p.i.), and the lung, liver and spleen were harvested and preserved in RNAlater® (Ambion) at −80°C until RNA extraction. All animal care and experimental procedures were conducted according to the guidelines for animal use in toxicology. The study protocol was approved by the Animal Care and Use Committee of the Shanghai Veterinary Research Institute, Chinese Academy of Agricultural Sciences.

### Total RNA isolation and microarray analysis

Total RNA extraction from tissues of Wistar rats was performed with the mirVana isolation kit (Ambion, USA), according to the manufacturer’s protocol. The quality and integrity of the RNA were measured using a Nanodrop-1000 and subsequently an Agilent 2100 Bioanalyzer (Agilent Technologies, USA). Only cases with RNA integrity numbers (RIN) ≥7–10 were used for further experiments. Briefly, the assay began using 5 μg of RNA from each sample, which was size fractionated using a YM-100 Microcon filter (Millipore, Bedford, MA, USA). The small RNAs (<300 nt) extracted were 3′ extended with poly (A) polymerase. miRNA microarrays following the miRbase v17.0 (including 1,096 miRNAs in the mouse, 679 in the rat, two in Chinese hamsters, and 55 control miRNA sequences) were used to analyze the expression profile of each sample. Briefly, hybridizations were performed according to the manual using μParaflo^@^ microfluidic technology (LC Sciences, USA). The Cy5 dye-labeled small RNAs (<300 nt) were dissolved in 100 μl 6 × SSPE buffer (0.90 M NaCl, 60 mM Na_2_HPO_4_, 6 mM EDTA, pH 6.8) containing 25% formamide at 34°C overnight. Hybridization images were scanned using a laser scanner (GenePix 4000B, Molecular Device) and digital analysis was performed using Array-Pro software (Media Cybernetics, Bethesda, MD). Microarray hybridizations were performed in duplicate for all samples. The data were normalized using a cyclic LOWESS (locally-weighted regression) method for further analysis.

Full details of the miRNA microarray were deposited in the Gene Expression Omnibus (GEO; http://www.ncbi.nlm.nih.gov/geo/) public database with the associated platform accession number GPL15710. The raw data are available through GEO with the series accession number GSE38802. The entire microarray data set was MIAME compliant. We defined the differentially expressed miRNA using the log2-fold changes in the ratio of the detected signals [log2 (infected/control)] and the Student’s *t*-test was used to calculate *P* values. The differentially expressed miRNAs were selected on the basis of a fold change >2 or < −2 and *P* values <0.05.

### Prediction of gene targets of differentially expressed miRNAs: Gene ontology, KEGG pathway analysis and miRNA–gene network analysis

The targets of the miRNAs were predicted using four online software packages: TargetScan, miRanda, PicTar, and RNAhybrid. The target genes of the differentially expressed miRNAs were analyzed in terms of their Gene Ontology (GO) categories and Kyoto Encyclopedia of Genes and Genomes (KEGG) pathways, using the DAVID (Database for Annotation, Visualization and Integrated Discovery) gene annotation tool [16]. The top 20% of the miRNA target genes were identified, and subjected to further miRNA–gene network analysis [17,18]. The relationship between the miRNAs and genes was evaluated by their differential expression values, and a miRNA–gene network was constructed according to the interactions of the miRNAs and genes in the Sanger miRNA database [19]. The adjacency matrix of miRNAs and genes (A = [ai,j]) was established on the basis of the relationship attributes between the genes and the miRNA, where ai,j shows the weight of the relation of gene i with miRNA j. In the diagram of the miRNA-gene network, a circle represents the gene and a square the miRNA, with the relationship between them represented by a line. Degrees in the center of the network represent the individual contribution of one miRNA or gene to the genes or miRNAs surrounding them. The key miRNAs and genes in the network usually have the highest degrees [20,21]. On the basis of the miRNA degree, the network that represented the crucial miRNAs and their targets could be established.

### Validation of microarray data with qPCR analysis

Differentially expressed miRNAs were validated using quantitative stem-loop reverse transcription RT-PCR (*qPCR*) with SYBR green. The stem-loop reverse transcription primers were designed following the method described by Chen *et al.*[22]*.* U87 RNA was selected as a housekeeping miRNA for normalization of the miRNA expression as previously reported [23]. The RNA templates for the qPCR were performed on the same samples used for microarray hybridizations. The primers for the qPCR experiment were optimized by the PCR analysis to evaluate the specificity and sensitivity. Total RNA from tissues was quantified using nanodrop-1000 and reverse-transcribed to cDNA using RT primers and a SuperScript™ III Reverse Transcriptase kit (Invitrogen, USA). The 25 μL qPCR reaction was as follows: 12.5 μL SYBR^@^ Premix Ex Taq™II (TaKaRa, Dalian, China), 1 μL of forward and reverse primer mixture, 1 μL cDNA template, 0.5 μL Rox Reference Dye II and 10 μL Easy Dilution. The cycling protocol was as follows: 95°C for 30 sec, followed by 40 cycles of 95°C for 5 sec and 60°C for 34 sec. The quantification of each miRNA relative to U87 was calculated using the 2^–△△Ct.^ method. All assays were performed in triplicate. The primer sequences are shown in Additional file 1: Table S1.

### Statistical analysis

Data are expressed as mean ± standard deviation (SEM). Differences between groups were determined by Student’s *t-*test, and statistical significance was reached at *P* ≤ 0.01.

## Results

### Differentially expressed miRNAs in tissues of infected Wistar rats

**Figure 1:**
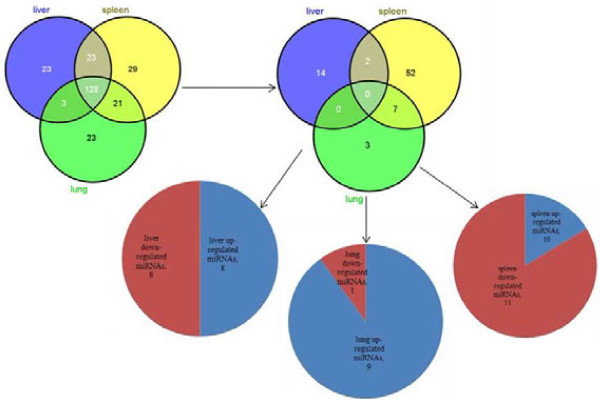
**Comparison of the observed differential expression miRNAs in liver, spleen and lung of Wistar rats before and 10 days after *S. japonicum *infection.**

In total, 1,777 mature miRNAs (Sanger miRbase v17.0) in the liver, spleen and lungs were profiles using miRNA microarrays. The miRNAs differentially expressed between infected and uninfected animals (with a signal intensity of >500) that were characterized in the different tissues are shown in Figure 1. Among these, 177, 201 and 175 miRNAs were detected in the liver, spleen and lung in Wistar rats before and 10 days post infection by *S.japonicum*. Differentially expressed miRNAs in Wistar rat*s* are listed separately in Additional file 2: Table S2, with values of log 2 (infected/control) and -fold changes. A positive log 2 value indicates up-regulation and a negative log 2 value indicates down-regulation. Among the detectable miRNAs that were differentially expressed in the samples from Wistar rats, a total of 78 different miRNA species were identified, of which 16 were in the liver (8 up regulated miRNAs and 8 down regulated miRNAs), 61 in the spleen (10 up regulated miRNAs and 51 down regulated miRNAs) and 10 in the lung (9 up regulated miRNAs and 1 down regulated miRNAs) (Figure 1). Examples of differentially expressed miRNAs in different tissues from Wistar rats before and 10d after *S japonicum* infection are presented in Table 1.

**Table 1:** **Examples of differential expressed miRNAs in Different tissues from Wistar rats before and 10d after*S. japonicum*infection**

**Tissue/Liver**	**Log2(infected/control)**	**Fold change**	**Tissue/Spleen**	**Log2(infected/control)**	**Fold change**
rno-miR-365*	2.08	4.23	rno-miR-204	2.29	4.89
rno-miR-29c	1.67	3.18	mmu-miR-720	1.94	3.84
mmu-miR-346*	1.24	2.36	rno-miR-27a	1.15	2.22
rno-miR-192*	1.23	2.35	rno-miR-29c	1.04	2.06
rno-miR-122*	1	2	rno-miR-223	1.02	2.03
mmu-miR-467a*	−1.1	0.47	mmu-miR-341*	−1.24	0.42
mmu-miR-467g	−1.45	0.37	rno-miR-483	−1.34	0.4
mmu-miR-467e*	−1.57	0.34	rno-miR-328a*	−1.57	0.34
mmu-miR-467c*	−1.59	0.33	rno-miR-19b	−1.59	0.33
rno-miR-451	−4.37	0.05	rno-miR-196c*	−1.66	0.32
Tissue/Lung	Log2(infected/control)	Fold change	mmu-miR-32*	−2.17	0.22
rno-miR-206	2.23	4.69	mmu-miR-328*	−2.63	0.16
rno-miR-223	1.82	3.53	rno-miR-32*	−2.85	0.14
rno-miR-98	1.2	2.3	mmu-miR-468	−3.49	0.09
mmu-miR-468	1.19	2.28	mmu-miR-691	−4.04	0.06
mmu-miR-669d	1.11	2.16	mmu-miR-297a	−4.62	0.04
rno-miR-328a*	1.1	2.14	mmu-miR-467h	−4.96	0.03
mmu-miR-494	−1.01	0.5	rno-miR-206	−5.08	0.03

### Analysis of the biological function of the differentially expressed miRNAs

The main functions of the differentially expressed miRNAs in the different tissues of Wistar rat*s* infected with *S. japonicum* are shown in Table 2, along with the reported functions. One differentially expressed miRNA in the liver has important functions in regulation of the expression of extracellular matrix proteins (miR-29c). Another differentially expressed miRNA this time in the host spleen had clear functions in regulation of adipocyte differentiation (miR-27a). Four differentially expressed miRNAs in the lung had functions in cell differentiation, protein expression and apoptosis, including promotion of muscle differentiation (miR-206), regulation of cholangiocyte expression factor (miR-98), targeting pro-apoptotic and antiapoptotic proteins (miR-494), myeloid lineage development and promoting granulocytic differentiation, and suppression of erythrocytic differentiation (miR-223).

**Table 2:** **Main functions of the differentially expressed miRNAs in different tissues in Wistar rats infected with*S. japonicum***

**miRNA**	**Function**	**PMID**
miR-206	Promoting muscle differentiation	16923828
miR-27a	Negative regulator of adipocyte differentiation	20060380
miR-29c	Regulating extracellular matrix proteins expression	18390668
miR-451	Erythroid differentiation; regulates the drug-transporter protein P-glycoprotein	20679397,20513743
miR-494	Targeting proapoptotic and antiapoptotic Proteins	20837890
miR-98	Regulating Cholangiocyte Expression of Cytokine-Inducible SHC protein	19592657
miR-223	Myeloid lineage development; promoting granulocytic differentiation, suppressing of erythrocytic differentiation	

### Prediction of targets of differentially expressed miRNAs

**Figure 2:**
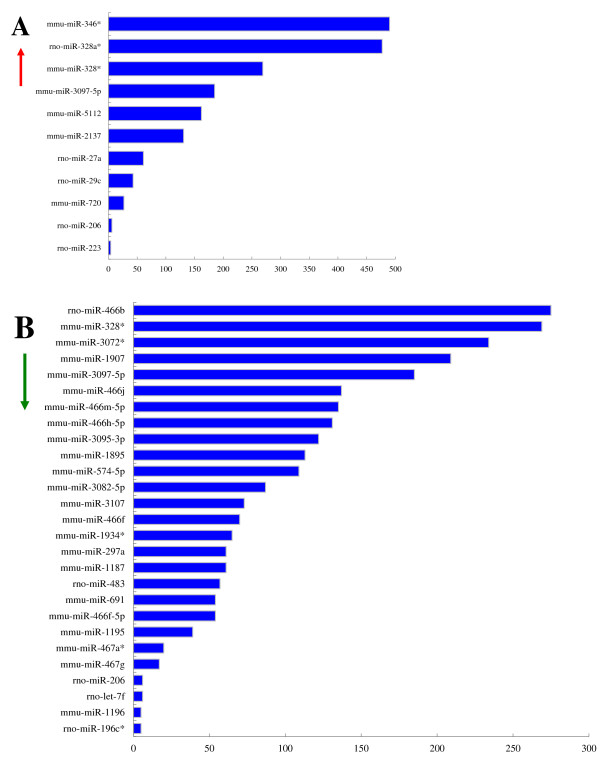
**The statistics of predicted target gene numbers for differentially expressed miRNA in Wistar rats following *S.japonicum *infection.** (**A**) Up-regulated miRNAs in Wistar rats were listed on the upper chart and (**B**) down-regulated miRNAs were in the lower chart. The vertical axis is the differentially expressed miRNAs and the horizontal axis is the target gene number.

We performed target prediction for all the differentially expressed miRNAs in the tissues of Wistar rats to identify the potential target mRNA(s). Of the 87 different miRNA species, only 38 produced predicted target genes using the three different online software programs. No differentially expressed miRNAs were detected among all three tissue types. The number of target genes predicted for each differentially expressed miRNA varied from 4 (miR-223) to 490 (miR-346*), with an average of 168 for up-regulated miRNAs and 96 for down-regulated miRNAs (Figure 2A and B).

### GO analyses of the predicted target genes of differentially expressed miRNAs

The GO analyses were performed on the target genes of differentially expressed miRNAs, and David gene annotation was applied to further explain the biological effect of miRNAs on the basis of the top 20% of the mRNA targets. As shown in Table 3, the top 18 enrichment GO annotations among the predicted target genes of differentially expressed miRNAs were identified. The GO analyses of these predicted target genes revealed that some of the target genes potentially had important biological functions in the host regarding defense against *S. japonicum* infection. The specific GO of the target genes related to the up-regulated expression of miRNAs in the liver, spleen and lungs of Wistar rats were involved mainly in biological processes (e.g. intracellular signaling cascade, phosphorylation), metabolic processes (e.g. negative regulation of macromolecule, phosphate, and phosphorus metabolic processes), cell components (e.g. cell projections, cytoskeleton, transcription factor complex), and molecular functions (e.g. cytoskeletal protein binding, identical protein binding, protein dimerization activity). The specific GO of the target genes related to the down-regulated miRNAs in the liver, spleen and lungs of Wistar rat*s* were involved mainly in biological processes (e.g. chordate embryonic development, homeostatic processes, ion transport, metal ion transport), cell components (e.g. cytoplasmic membrane-bound vesicle, cytoplasmic vesicle, endomembrane system, endosome, Golgi apparatus), and molecular functions (e.g. channel activity, nucleotide binding, passive transmembrane transporter activity).

**Table 3:** **GO analysis of the target genes of the differentially expressed miRNAs in different tissues of Wistar rats following*S. japonicum*infection**

**Up-regulated**	**Down-regulated**
**Biological process**	
Intracellular signaling cascade	Chordate embryonic development
Negative regulation of macromolecule metabolic process	Embryonic development ending in birth or egg hatching
Phosphate metabolic process	Homeostatic process
Phosphorus metabolic process	Metal ion transport
Phosphorylation	Positive regulation of transcription from RNA polymerase II promoter
**Cell component**	
Cell projection part	Cytoplasmic membrane-bounded vesicle
Cytoskeletal part	Cytoplasmic vesicle
Cytoskeleton	Endomembrane system
Extrinsic to membrane	Endosome
Intracellular non-membrane-bounded organelle	Golgi apparatus
Membrane fraction	Internal side of plasma membrane
Microtubule	Intrinsic to plasma membrane
Microtubule cytoskeleton	Membrane-bounded vesicle
Non-membrane-bounded organelle	Postsynaptic membrane
Transcription factor complex	Vesicle
**Molecular function**	
Cytoskeletal protein binding	Channel activity
Identical protein binding	Nucleotide binding
Protein dimerization activity	Passive transmembrane transporter activity

### KEGG pathway analyses of the predicted target genes of differentially expressed miRNAs

The KEGG pathway annotation of all the target genes of the miRNAs is shown in Table 4. Among the target genes of the differentially expressed miRNAs in the liver of Wistar rats, miR-346* has an important role in induction of the MAPK signaling pathway. Among the targets for differentially expressed miRNAs in the spleen of Wistar rats, miR-3584-5p, miR-328*, miR-3095-3p and miR-3072* have important roles in signal pathway induction (involved in the Wnt, MAPK, mTOR, and neurotrophin signaling pathways) and immune regulation (involved in the chemokine signaling pathway in miR-1895).

**Table 4:** **KEGG analysis of the target genes of the differentially expressed miRNAs in different tissues of Wistar rats following*S.japonicum*infection**

**Category**	**Term**	**Genes**	**P value**
mmu-miR-346*	Focal adhesion	CAV1, PDGFB, FLT4, ACTN1, ITGA3, COL5A3, PPP1CB, HRAS1, AKT1, LAMA4, CCND2, ITGB8, GSK3B, COL6A2, PDGFRB, COL1A1, PARVB, DIAP1	2.37E-04
	MAPK signaling pathway	FGFR4, PDGFB, CACNB1, MKNK2, TGFB3, CACNG2, CACNG1, SRF, CACNA2D2, HRAS1, AKT1, DUSP3, RPS6KA2, IKBKG, PPP3CC, PDGFRB, PRKACB, TRAF6, MAP2K6	2.38E-03
	Long-term potentiation	ADCY1, RPS6KA2, CALM3, PPP3CC, PRKACB, CAMK2A, PPP1CB, HRAS1	8.39E-03
rno-miR-3584-5p	Wnt signaling pathway	PPP2R1B, PPP2R5B, APC2, MAP3K7, SFRP5, RAC2, PRICKLE1, NFAT5, LRP6, CAMK2B, RHOC, PPP2R5E, NFATC2, FOSL1, WNT8B	4.27E-03
	Neurotrophin signaling pathway	IRAK2, PDK1, MAPK11, IRS1, YWHAE, TP73, MAP3K3, SORT1, RAP1A, CAMK2B, RHOC, NGFR, ARHGDIB	8.81E-03
	Axon guidance	NRP1, EFNB3, DPYSL5, EPHB1, EPHB6, RAC2, SEMA4G, NFAT5, SEMA3B, RHOC, EFNA4, NFATC2, SEMA4A	9.36E-03
rno-miR-328*	MAPK signaling pathway	TAOK1, TGFBR1, RELA, CACNB1, MKNK2, TGFB3, MKNK1, CACNG2, CACNG1, AKT1, DUSP3, MAP3K3, DUSP14, RPS6KA2, PPP3CB, MAPK9, PDGFRB, CACNA1E, RASA1, MAP3K11	3.52E-08
	Dilated cardiomyopathy	ADCY1, ADCY9, ITGA5, TGFB3, CACNB1, CACNG2, CACNG1	3.79E-03
	Focal adhesion	AKT1, CCND2, ITGA5, FLT4, COL6A2, PDGFRB, MAPK9, COL5A3, PARVB, DIAP1	4.87E-03
	Axon guidance	ABLIM2, NRAS, PAK7, SEMA4G, LIMK2, EFNB1, NFAT5, L1CAM, PAK1, CHP, EPHB3, SRGAP2	1.02E-05
	T cell receptor signaling pathway	NRAS, PAK7, RASGRP1, PIK3CD, NFAT5, MAPK9, PAK1, CHP	2.68E-03
	Wnt signaling pathway	WNT1, CCND1, PRICKLE1, VANGL2, LRP6, NFAT5, MAPK9, CHP, FZD4	3.48E-03
	Neurotrophin signaling pathway	IRAK1, NRAS, IRS3, NTF4, PIK3CD, MAPK9, FRS2, PRKCD	5.99E-03
mmu-miR-466j	Cell adhesion molecules (CAMs)	CD274, CD4, CDH1, CDH2, CD28, CLDN15	8.82E-03
mmu-miR-1895	Chemokine signaling pathway	BRAF, TIAM1, RHOA, GRK6, JAK2, SHC2	4.55E-03
mmu-miR-3095-3p	mTOR signaling pathway	RPS6KA2, ULK2, PIK3R5, RPTOR	6.53E-03
mmu-miR-3072*	Neurotrophin signaling pathway	PDK1, IRAK3, RPS6KA1, RPS6KA2, GRB2, MAPK14, PIK3R5, SHC2, HRAS1	4.12E-04
	Fc epsilon RI signaling pathway	PDK1, GRB2, MAPK14, IL13, PIK3R5, HRAS1	5.48E-03
	Focal adhesion	VEGFB, CCND2, GRB2, COMP, COL6A2, PIK3R5, SHC2, MYLK, HRAS1	6.01E-03
	mTOR signaling pathway	VEGFB, RPS6KA1, RPS6KA2, RPS6KB2, PIK3R5	6.53E-03
mmu-miR-5112	Melanogenesis	GNAQ, ADCY8, CREB1, PRKACA, WNT9A, PRKACB	2.34E-03

### MiRNA–gene network

**Figure 3:**
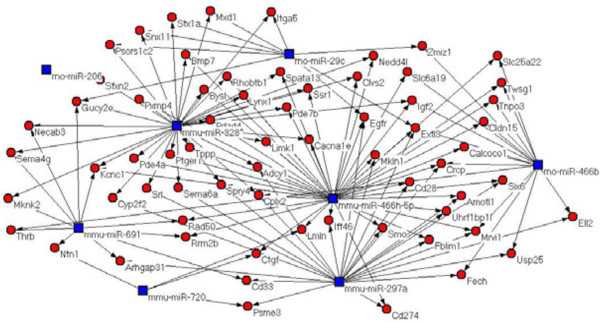
**microRNA-target-network.** Red cycle nodes represent mRNA and blue box nodes represent miRNA. Significant miRNAs differentially expressed in different tissues of rat. Edges represent the inhibitive effect of microRNA on mRNA.

As shown in Figure 3, we carried out miRNA–gene network analysis to study the relationships among the differentially expressed miRNAs in the different tissues from Wistar rats. We established miRNA regulatory networks in schistosome infection of Wistar rats, which indicated that the differentially expressed miRNA target genes might have important roles in the response of the host to *S. japonicum* infection.

### Validation of miRNA microarray data with qPCR analysis

**Figure 4:**
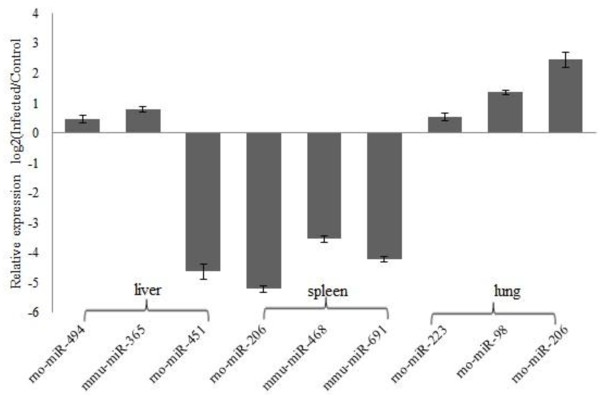
***qPCR *confirmation of miRNA microarray data subset.** Selected miRNA expression profile was validated in liver, spleen and lung between Wistar rats with *qPCR*. Expression rates between various samples are showed by fold change. The data presents the mean and standard error of the mean derived from triplicate experiments.

Nine selected miRNAs and the housekeeping miRNA miR-U87 were assayed by *qPCR* to confirm the findings of the expression profiles detected by the microarray platform. It was found that miR-494, miR-365 and miR-451 were present in liver, miR-206, miR-468 and miR-691 in spleen, and miR-223, miR-98 and miR-206 in lung. The results showed that the expression patterns detected by *qPCR* correlated well with the microarray data (Figure 4, Table 1).

## Discussion

More than 40 species of mammal, including cattle, sheep, goats, rabbits, mice, water buffalo, pigs and rats, can be infected by *S. japonicum*. Most of these species are susceptible to the infection, while some species, such as the water buffalo, pig and rat, are less susceptible, as shown by lower parasite development rates and smaller size of adult worms [4,24]. The rat is reported to be a model of a semi-permissive host. Only a small percentage of infecting cercariae were found to develop into mature adults in the portal mesenteric veins of Wistar rats 5 weeks post infection, and most schistosomes fail to complete their life cycle, as shown by the finding of small eggs in the feces during the 6 weeks post-infection [4]. However, knowledge of the molecular mechanisms underlying this phenomenon remains incomplete, and multiple factors are thought to be involved in the process.

The juvenile schistosomulum is an important stage in the intra-mammalian phase of the schistosome lifecycle, and represents a key target for elimination of infection by both natural and vaccine-induced host immune response. After penetrating the host skin, schistosomula move to the lungs around 3 days post cercarial exposure, then migrate to the liver. Most worms reach the hepatic portal system approximately 10 days post infection and remain there during sexual maturation and pairing. During this period, the schistosomulum undergoes rapid development, and must also respond to immune attack from the host [25]. Schistosome infection stimulates the host response, which results in changes in various host factors that may regulate the survival and development of early schistosomula [26]. These important features of the schistosome life cycle dictate the selection of 10 day post-cercarial infections for future study.

MicroRNAs have been identified as a class of naturally occurring single-stranded short non-coding RNAs, which consist of 21–23 nucleotides that exist in a wide range of eukaryotic organisms [27]. Given the spatial and temporal differential expression model of miRNAs, and their conservation across a wide range of species, miRNAs are believed to play similar central roles in all mammalian cells by preventing the translation of downstream target mRNAs, and ultimately inhibiting the expression of multiple genes [28]. In the current study, we have identified the specific miRNAs that may be involved in the pathophysiological processes of schistosome infection in Wistar rats. Most of the host miRNAs in different tissues are expressed in both uninfected and infected Wistar rats, however, some differentially expressed miRNAs were also characterized that were expected to play important roles in the pathology of the semi-permissive host model. Our results strongly suggest a regulatory role for host miRNAs in response to an active schistosome infection, and provide a better understanding of the interplay between the schistosome and its definitive host, and the host response.

Live schistosomes can persist very effectively in some definitive hosts, which means that schistosomes have adapted to these host environments and can evade the host immune response [29]. Understanding the nature of the host responses induced by schistosome infection within different tissues, will demonstrate how they may interact with their host to facilitate survival and establish a long-term infection [30]. MiRNAs are reported to be important regulators of the immune system through the regulation of specific immune functions [31]. Some miRNAs such as miR-223, were found to be highly expressed in the lungs of the Wistar rats, potentially acting as immune regulators of the host immune response. MiR-223 has been shown to be an essential modulator of myeloid and to mediate the development of the myeloid lineage. Specific genes with immunoregulatory functions may act to control the magnitude of the inflammatory response caused by schistosome infection [30,31].

MiRNAs may also regulate, growth, differentiation, and metabolism [32,33]. Schistosomes require key nutrient molecules, such as fatty acids, sterols, purines, nine essential amino acids, arginine and tyrosine, from the host because they are unable to synthesize them [34]. The differential expression of genes related to nutrition, metabolism and protein expression in host Wistar rats, regulated via miRNAs, may result in the abnormal development of the worms. As shown in this study, miR-27a was up-regulated in the spleens of infected Wistar rats, which indicates a lower level of regulation of adipocyte differentiation in the host [35]. MiR-451 was differentially expressed in the livers of infected animals, which suggests that erythroid differentiation may be altered following *S. japonicum* infection [36,37]. Various other miRNAs, such as miR-29c and miR-98, were differentially expressed in the spleen and lungs of the infected animals respectively. These miRNAs potentially function in suppression of extracellular matrix protein expression and cholangiocyte expression of cytokine-inducible SHC protein [38-40]. However, the biological functions of these proteins in the schistosome-infected host are still unclear and further study is needed. To our surprise, miR-494 (which targets proapoptotic and antiapoptotic proteins) was differentially expressed in the lungs of the infected animals [36,41]. Research has shown that schistosome infection can result in the production of certain factors that regulate apoptosis of host immune cells [42]. Our results indicate that miRNAs may participate in the apoptosis pathway in schistosome-infected animals.

The GO analysis of the differentially expressed miRNAs, was based on reported and predicted target genes, and showed that a high-enrichment of GOs were involved in the metabolism of phosphorus and phosphate, phosphorylation, metal ion transport, and channel activity. Phosphorylation of schistosomal proteins has been reported to be involved in schistosome development and growth [43]. The regulation of the host tissue phosphorylation pathway by miRNAs may also regulate the phosphorylation level of parasite proteins, and thus affect the development of the worms. The KEGG pathway analysis of the target genes of the differentially expressed miRNAs showed that the genes were involved mainly in immune-related pathways, such as the Toll-like receptor and chemokine signaling pathways, and in signal induction pathways, such as the MAPK and Wnt signaling pathways.

## Conclusions

In conclusion, this study presents for the first time an extensive analysis of miRNA expression profiles in the liver, lungs and spleen of schistosome-infected Wistar rats, a semi-permissive host. The findings of this study provide novel miRNA-based information that increases our understanding of the pathophysiological processes involved in *S. japonicum* infection in Wistar rats, and provides potential targets for future schistosome control strategies.

## Competing interests

The authors have declared that no competing interests.

## Authors’ contributions

Conceived and designed the experiments: JL, HH, and JP. Performed the experiments and analysed the data: HH, JP, YH, MZ, YHH, ZF, YS. Wrote and revised the manuscript: HH, JP, JX, JT and JL. All authors read and approved the final manuscript.

## Supplementary Material

Additional file 1: Table S1 Sequences of the primers used for stem-loop RT-PCR. Click here for file

Additional file 2: Table S2 miRNAs expression profile in different tissues of Wistar rats before and 10 days post infected with *S.japonicum.* Click here for file
